# Supplementary figures and images for: Prognostic nomogram for uncontrolled type 2 diabetes using Thailand nation-wide cross-sectional studies
Source: PLoS One. 2024 Apr 10;19(4):e0298010. doi: 10.1371/journal.pone.0298010 (PMC11006157; doi:10.1371/journal.pone.0298010)

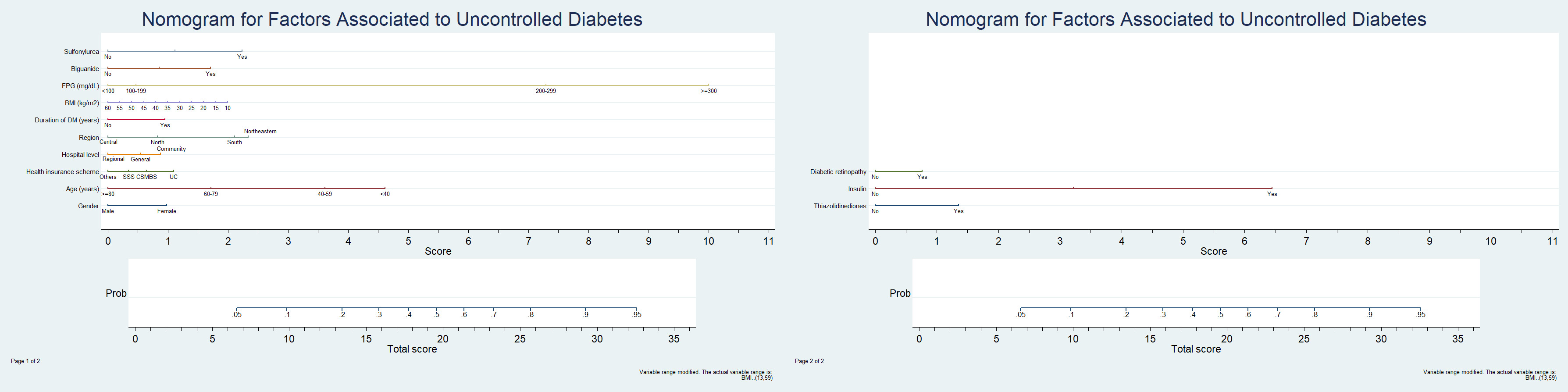

Supplement: S1 Fig — Nomogram included gender, age group, health insurance scheme, hospital level, regions of Thailand, duration of diabetes, body mass index (BMI), fasting plasma glucose (FPG) level, biguanide prescription, sulfonylurea prescription, thiazolidinedione prescription, insulin injection, diagnosis of diabetes. The nomogram is valued to obtain the probability of uncontrolled diabetes by adding up the points identified on the points scale for each variable. UC: universal coverage, SSS: social security scheme, CSMBS: civil servant medical benefit scheme, BMI: body mass index, FPG: fasting plasma glucose, HbA1c: hemoglobin A1c. (TIF) [file pone.0298010.s001.tif]

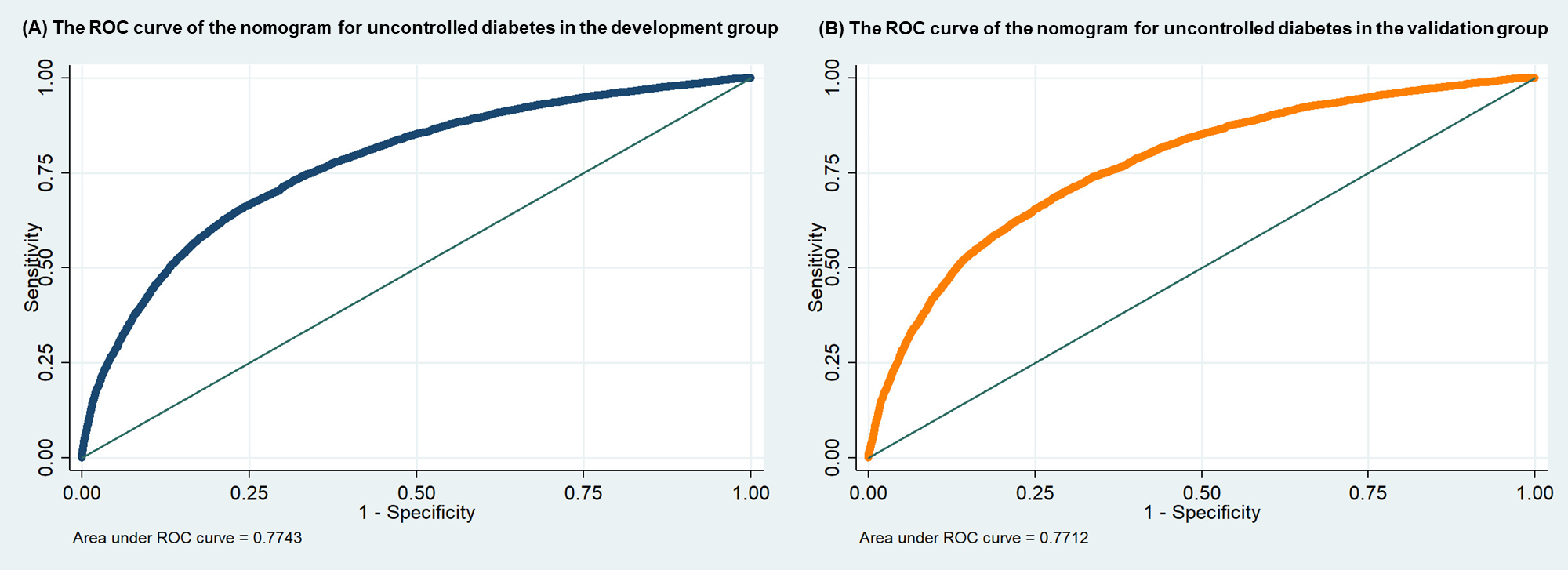

Supplement: S2 Fig — The ROC curves of the nomogram for uncontrolled diabetes in the development group (A) and validation group (B). (TIF) [file pone.0298010.s002.tif]

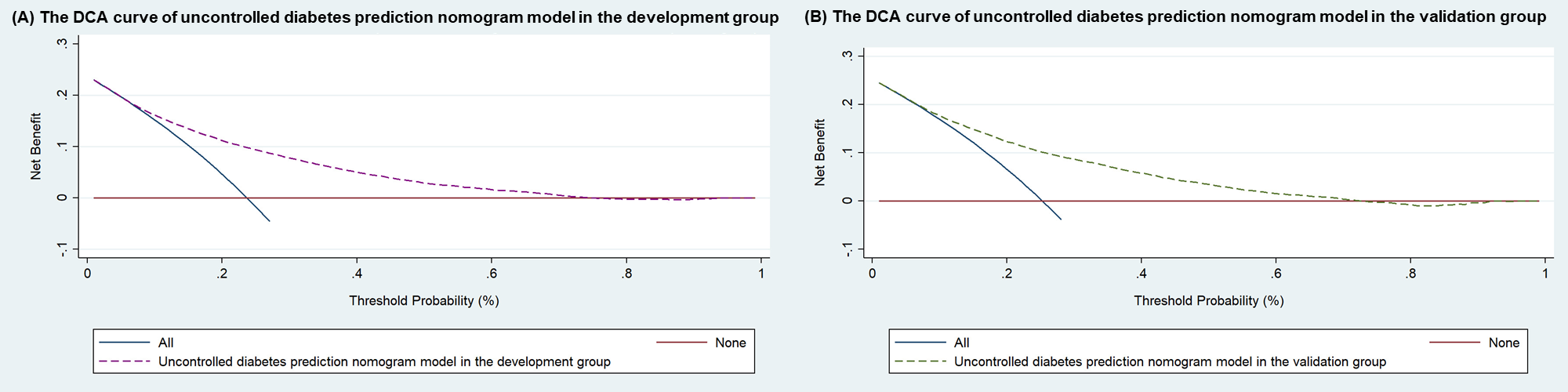

Supplement: S3 Fig — The DCA of the prediction nomogram model in the development group (A) and validation group (B). (TIF) [file pone.0298010.s003.tif]
