# Supplementary material for: Prognostic nomogram for uncontrolled type 2 diabetes using Thailand nation-wide cross-sectional studies
Source: PLoS One. 2024 Apr 10;19(4):e0298010. doi: 10.1371/journal.pone.0298010 (PMC11006157; doi:10.1371/journal.pone.0298010)
Supplement: S1 Table — SD: standard deviation, UC: universal coverage, SSS: social security scheme, CSMBS: civil servant medical benefit scheme, FPG: fasting plasma glucose, HbA1c: hemoglobin A1c, mg/dL: milligrams per deciliter. (DOCX) [file pone.0298010.s004.docx]

| **Table 1. Comparison of demographic characteristics between the development group and validation group.** | | | |
| --- | --- | --- | --- |
| **Characteristics** | **Development Group** | **Validation Group** | **P-value** |
|  | **2018** | **2015** |  |
|  | **N=38,568** | **N=32,616** |  |
|  | **n (%)** | **n (%)** |  |
| **Gender** |  |  | 0.123 |
| Male | 12,748 (33.05) | 10,603 (32.51) |  |
| Female | 25,820 (66.95) | 22,013 (67.49) |  |
| **Age (years)** |  |  |  |
| Mean ± SD | 62.34 ± 11.00 | 61.50 ± 10.96 | <0.001 |
| <40 | 786 (2.04) | 777 (2.38) | <0.001 |
| 40-49 | 3,874 (10.04) | 3,683 (11.29) |  |
| 50-59 | 10,645 (27.60) | 9,420 (28.88) |  |
| 60-69 | 13,334 (34.57) | 10,965 (33.62) |  |
| ≥70 | 9,929 (25.74) | 7,771 (23.83) |  |
| **Health insurance scheme** |  |  | <0.001 |
| UC | 30,269 (78.48) | 24,905 (76.36) |  |
| CSMBS | 6,270 (16.26) | 5,716 (17.53) |  |
| SSS | 1,523 (3.95) | 1,335 (4.09) |  |
| Others | 506 (1.31) | 660 (2.02) |  |
| **Regions** |  |  | <0.001 |
| North | 8,920 (23.13) | 5,133 (15.74) |  |
| Central | 12,505 (32.42) | 13,410 (41.11) |  |
| Northeast | 10,610 (27.51) | 9,444 (28.96) |  |
| South | 6,533 (16.94) | 4,629 (14.19) |  |
| **Hospital level** |  |  | <0.001 |
| Regional Hospitals | 2,670 (6.92) | 2,919 (8.95) |  |
| General Hospitals | 7,554 (19.59) | 7,838 (24.03) |  |
| Community Hospitals | 28,344 (73.49) | 21,859 (67.02) |  |
| **Hypertension** |  |  | 0.593 |
| No | 8,455 (21.92) | 7,096 (21.76) |  |
| Yes | 30,113 (78.08) | 25,520 (78.24) |  |
| **Biguanide** |  |  | <0.001 |
| No | 9,396 (24.36) | 9,084 (27.85) |  |
| Yes | 29,172 (75.64) | 23,532 (72.15) |  |
| **Sulfonylurea** |  |  | <0.001 |
| No | 15,800 (40.97) | 12,195 (37.39) |  |
| Yes | 22,768 (59.03) | 20,421 (62.61) |  |
| **Thiazolidinediones** |  |  | <0.001 |
| No | 33,865 (87.81) | 29,269 (89.74) |  |
| Yes | 4,703 (12.19) | 3,347 (10.26) |  |
| **Insulin** |  |  | 0.032 |
| No | 30,109 (78.07) | 25,243 (77.39) |  |
| Yes | 8,459 (21.93) | 7,373 (22.61) |  |
| **Diabetic retinopathy** |  |  | <0.001 |
| No | 36,624 (94.96) | 30,573 (93.74) |  |
| Yes | 1,943 (5.04) | 2,043 (6.26) |  |
| **FPG (mg/dL)** |  |  |  |
| Mean ± SD | 153.53 ± 54.29 | 153.89 ± 55.76 | 0.400 |
| **HbA1c (%)** |  |  | <0.001 |
| <9 | 25,363 (76.00) | 19,682 (74.72) |  |
| ≥9 | 8,010 (24.00) | 6,659 (25.28) |  |

SD: standard deviation, UC: universal coverage, SSS: social security scheme, CSMBS: civil servant medical benefit scheme, FPG: fasting plasma glucose, HbA1c: hemoglobin A1c, mg/dL: milligrams per deciliter
