# Supplementary material for: Prognostic nomogram for uncontrolled type 2 diabetes using Thailand nation-wide cross-sectional studies
Source: PLoS One. 2024 Apr 10;19(4):e0298010. doi: 10.1371/journal.pone.0298010 (PMC11006157; doi:10.1371/journal.pone.0298010)
Supplement: S2 Table — SD: standard deviation, BMI: body mass index, UC: universal coverage, SSS: social security scheme, CSMBS: civil servant medical benefit scheme, FPG: fasting plasma glucose, HbA1c: hemoglobin A1c, SGLT2 inhibitors: sodium-glucose Cotransporter-2 Inhibitors, mg/dL: milligrams per deciliter, kg/m2: kilogram per square meter, kg: kilogram, cm: centimeter. (DOCX) [file pone.0298010.s005.docx]

| **Table 2. Univariable logistic regression analysis in the development group (n=38,568).** | | | | | |
| --- | --- | --- | --- | --- | --- |
| **Characteristics** | **HbA1c** | | **Crude**  **Odds ratio** | **95% CI** | **P-value** |
|  | **<9** | **≥9** |  |  |  |
|  | **n (%)** | **n (%)** |  |  |  |
| **Gender** |  |  |  |  |  |
| Male | 8,740 (34.46) | 2,289 (28.58) | 1 |  |  |
| Female | 16,623 (65.54) | 5,721 (71.42) | 1.31 | 1.24-1.39 | <0.001 |
| **Hospital level** |  |  |  |  |  |
| Regional Hospitals | 1,930 (7.61) | 565 (7.05) | 1 |  |  |
| General Hospitals | 5,087 (20.06) | 1,498 (18.7) | 1.01 | 0.90-1.12 | 0.916 |
| Community Hospitals | 18,346 (72.33) | 5,947 (74.24) | 1.11 | 1.00-1.22 | 0.042 |
| **Age group (years)** |  |  |  |  |  |
| <40 | 417 (1.64) | 254 (3.17) | 1 |  |  |
| 40-59 | 8,735 (34.44) | 3,822 (47.72) | 0.72 | 0.61-0.84 | <0.001 |
| 60-79 | 14,411 (56.82) | 3,675 (45.88) | 0.42 | 0.36-0.49 | <0.001 |
| ≥80 | 1,800 (7.10) | 259 (3.23) | 0.24 | 0.19-0.29 | <0.001 |
| **Duration of diabetes (years)** |  |  |  |  |  |
| 0-9 | 13,459 (58.37) | 3,460 (47.94) | 1 |  |  |
| 10-19 | 8,656 (37.54) | 3,315 (45.93) | 1.49 | 1.41-1.57 | <0.001 |
| 20-29 | 838 (3.63) | 373 (5.17) | 1.73 | 1.52-1.97 | <0.001 |
| ≥30 | 105 (0.46) | 69 (0.96) | 2.56 | 1.88-3.47 | <0.001 |
| **Height (cm)** |  |  |  |  |  |
| Mean ± SD | 158.03 ± 8.20 | 157.63 ± 7.97 | 0.99 | 0.99-1.00 | <0.001 |
| **Weight (kg)** |  |  |  |  |  |
| Mean ± SD | 64.37 ± 13.48 | 64.35 ± 13.49 | 1 | 1.00-1.00 | 0.921 |
| **Waist (cm)** |  |  |  |  |  |
| Mean ± SD | 88.85 ± 10.99 | 88.92 ± 11.14 | 1 | 1.00-1.00 | 0.618 |
| **BMI group (kg/m^2^)** |  |  |  |  |  |
| Mean ± SD | 25.72 ± 4.75 | 25.86 ± 4.81 | 1.01 | 1.00-1.01 | 0.024 |
| 18.50-22.99 | 6,262 (24.95) | 1,918 (24.18) | 1 |  |  |
| 23.00-24.99 | 5,008 (19.96) | 1,485 (18.72) | 0.97 | 0.90-1.05 | 0.411 |
| 25.00-29.99 | 8,790 (35.03) | 2,862 (36.08) | 1.06 | 0.99-1.14 | 0.071 |
| ≥30.00 | 4,073 (16.23) | 1,362 (17.17) | 1.09 | 1.01-1.18 | 0.031 |
| <18.50 | 961 (3.83) | 306 (3.86) | 1.04 | 0.91-1.19 | 0.583 |
| **Regions** |  |  |  |  |  |
| North | 5,977 (23.57) | 1,722 (21.50) | 1 |  |  |
| Central | 9,096 (35.86) | 2,081 (25.98) | 0.79 | 0.74-0.85 | <0.001 |
| Northeastern | 6,002 (23.66) | 2,561 (31.97) | 1.48 | 1.38-1.59 | <0.001 |
| South | 4,288 (16.91) | 1,646 (20.55) | 1.33 | 1.23-1.44 | <0.001 |
| **Health insurance scheme** |  |  |  |  |  |
| UC | 19,499 (76.88) | 6,516 (81.35) | 1 |  |  |
| CSMBS | 4,526 (17.84) | 1,074 (13.41) | 0.71 | 0.66-0.76 | <0.001 |
| SSS | 1,013 (3.99) | 325 (4.06) | 0.96 | 0.84-1.09 | 0.533 |
| Others | 325 (1.28) | 95 (1.19) | 0.87 | 0.69-1.10 | 0.255 |
| **Hypertension** |  |  |  |  |  |
| No | 5,072 (20.00) | 2,110 (26.34) |  |  |  |
| Yes | 20,291 (80.00) | 5,900 (73.66) | 0.70 | 0.66-0.74 | <0.001 |
| **Dyslipidemia** |  |  |  |  |  |
| No | 7,255 (28.60) | 2,182 (27.24) |  |  |  |
| Yes | 18,108 (71.40) | 5,828 (72.76) | 1.07 | 1.01-1.13 | 0.018 |
| **Gout** |  |  |  |  |  |
| No | 23,815 (93.90) | 7,696 (96.08) |  |  |  |
| Yes | 1,548 (6.10) | 314 (3.92) | 0.63 | 0.55-0.71 | <0.001 |
| **Renal insufficiency** |  |  |  |  |  |
| No | 21,060 (83.03) | 6,771 (84.53) |  |  |  |
| Yes | 4,303 (16.97) | 1,239 (15.47) | 0.90 | 0.84-0.96 | 0.002 |
| **Diabetic kidney diseases** |  |  |  |  |  |
| No | 23,559 (92.89) | 7,417 (92.60) |  |  |  |
| Yes | 1,804 (7.11) | 593 (7.40) | 1.04 | 0.95-1.15 | 0.38 |
| **Diabetic retinopathy** |  |  |  |  |  |
| No | 24,194 (95.39) | 7,440 (92.88) | 1 |  |  |
| Yes | 1,168 (4.61) | 570 (7.12) | 1.59 | 1.43-1.76 | <0.001 |
| **Cerebrovascular accident** |  |  |  |  |  |
| No | 24,522 (96.68) | 7,797 (97.34) |  |  |  |
| Yes | 841 (3.32) | 213 (2.66) | 0.8 | 0.68-0.93 | 0.003 |
| **Coronary artery disease** |  |  |  |  |  |
| No | 24,287 (95.76) | 7,717 (96.34) |  |  |  |
| Yes | 1,076 (4.24) | 293 (3.66) | 0.86 | 0.75-0.98 | 0.022 |
| **Biguanide** |  |  |  |  |  |
| No | 6,012 (23.70) | 1,804 (22.52) | 1 |  |  |
| Yes | 19,351 (76.30) | 6,206 (77.48) | 1.07 | 1.01-1.14 | 0.029 |
| **Sulfonylurea** |  |  |  |  |  |
| No | 10,751 (42.39) | 2,813 (35.12) | 1 |  |  |
| Yes | 14,612 (57.61) | 5,197 (64.88) | 1.36 | 1.29-1.43 | <0.001 |
| **Thiazolidinediones** |  |  |  |  |  |
| No | 22,391 (88.28) | 6,718 (83.87) | 1 |  |  |
| Yes | 2,972 (11.72) | 1,292 (16.13) | 1.45 | 1.35-1.56 | <0.001 |
| **Insulin** |  |  |  |  |  |
| No | 21,580 (85.08) | 4,563 (56.97) | 1 |  |  |
| Yes | 3,783 (14.92) | 3,447 (43.03) | 4.31 | 4.07-4.56 | <0.001 |
| **SGLT2 Inhibitors** |  |  |  |  |  |
| No | 25,304 (99.77) | 7,988 (99.73) |  |  |  |
| Yes | 59 (0.23) | 22 (0.27) | 1.18 | 0.72-1.93 | 0.506 |
| **Calcium channel blockers** |  |  |  |  |  |
| No | 14,631 (57.69) | 5,265 (65.73) |  |  |  |
| Yes | 10,732 (42.31) | 2,745 (34.27) | 0.71 | 0.67-0.75 | <0.001 |
| **RAS blockers** |  |  |  |  |  |
| No | 10,716 (42.25) | 3,672 (45.84) |  |  |  |
| Yes | 14,647 (57.75) | 4,338 (54.16) | 0.86 | 0.82-0.91 | <0.001 |
| **Beta-blocker** |  |  |  |  |  |
| No | 20,953 (82.61) | 6,884 (85.94) |  |  |  |
| Yes | 4,410 (17.39) | 1,126 (14.06) | 0.78 | 0.72-0.83 | <0.001 |
| **Diuretics** |  |  |  |  |  |
| No | 21,721 (85.64) | 7,069 (88.25) |  |  |  |
| Yes | 3,641 (14.36) | 941 (11.75) | 0.79 | 0.74-0.86 | <0.001 |
| **Statins** |  |  |  |  |  |
| No | 7,683 (30.29) | 2,325 (29.03) |  |  |  |
| Yes | 17,680 (69.71) | 5,685 (70.97) | 1.06 | 1.01-1.12 | 0.031 |
| **FPG (mg/dL)** |  |  |  |  |  |
| Mean ± SD | 141.76 ± 40.34 | 187.96 ± 71.50 | 1.02 | 1.02-1.02 | <0.001 |
| <100 | 2,123 (8.74) | 460 (6.04) | 1 |  |  |
| 100-199 | 20,429 (84.11) | 4,414 (57.93) | 1 | 0.90-1.11 | 0.958 |
| 200-299 | 1,568 (6.46) | 2,218 (29.11) | 6.53 | 5.79-7.36 | <0.001 |
| ≥300 | 167 (0.69) | 527 (6.92) | 14.56 | 11.91-17.81 | <0.001 |

SD: standard deviation, BMI: body mass index, UC: universal coverage, SSS: social security scheme, CSMBS: civil servant medical benefit scheme, FPG: fasting plasma glucose, HbA1c: hemoglobin A1c, SGLT2 inhibitors: sodium-glucose Cotransporter-2 Inhibitors, mg/dL: milligrams per deciliter, kg/m2: kilogram per square meter, kg: kilogram, cm: centimeter
