# Supplementary material for: Prognostic nomogram for uncontrolled type 2 diabetes using Thailand nation-wide cross-sectional studies
Source: PLoS One. 2024 Apr 10;19(4):e0298010. doi: 10.1371/journal.pone.0298010 (PMC11006157; doi:10.1371/journal.pone.0298010)
Supplement: S3 Table — SD: standard deviation, UC: universal coverage, SSS: social security scheme, CSMBS: civil servant medical benefit scheme, BMI: body mass index, FPG: fasting plasma glucose, HbA1c: hemoglobin A1c, mg/dL: milligrams per deciliter, kg/m2: kilogram per square meter. (DOCX) [file pone.0298010.s006.docx]

| **Table 3. Multivariable logistic regression analysis for factors associated with uncontrolled diabetes in the development group (n=38,568).** | | | | | |
| --- | --- | --- | --- | --- | --- |
| **Characteristics** | **HbA1c** | | **Adjusted**  **Odds ratio** | **95% CI** | **P-value** |
|  | **<9** | **≥9** |  |  |  |
|  | **n (%)** | **n (%)** |  |  |  |
| **Gender** |  |  |  |  |  |
| Male | 8,740 (34.46) | 2,289 (28.58) | 1 |  |  |
| Female | 16,623 (65.54) | 5,721 (71.42) | 1.25 | 1.17-1.34 | <0.001 |
| **Age group (years)** |  |  |  |  |  |
| <40 | 417 (1.64) | 254 (3.17) | 1 |  |  |
| 40-59 | 8,735 (34.44) | 3,822 (47.72) | 0.80 | 0.65-0.97 | 0.025 |
| 60-79 | 14,411 (56.82) | 3,675 (45.88) | 0.52 | 0.42-0.63 | <0.001 |
| ≥80 | 1,800 (7.10) | 259 (3.23) | 0.35 | 0.27-0.45 | <0.001 |
| **Health insurance scheme** |  |  |  |  |  |
| UC | 19,499 (76.88) | 6,516 (81.35) | 1 |  |  |
| CSMBS | 4,526 (17.84) | 1,074 (13.41) | 0.90 | 0.83-0.99 | 0.024 |
| SSS | 1,013 (3.99) | 325 (4.06) | 0.84 | 0.72-0.99 | 0.037 |
| Others | 325 (1.28) | 95 (1.19) | 0.78 | 0.59-1.04 | 0.088 |
| **Hospital level** |  |  |  |  |  |
| Regional Hospitals | 1,930 (7.61) | 565 (7.05) | 1 |  |  |
| General Hospitals | 5,087 (20.06) | 1,498 (18.70) | 1.13 | 0.99-1.30 | 0.079 |
| Community Hospitals | 18,346 (72.33) | 5,947 (74.24) | 1.22 | 1.08-1.38 | 0.002 |
| **Regions** |  |  |  |  |  |
| North | 5,977 (23.57) | 1,722 (21.50) | 1 |  |  |
| Central | 9,096 (35.86) | 2,081 (25.98) | 0.83 | 0.76-0.90 | <0.001 |
| Northeastern | 6,002 (23.66) | 2,561 (31.97) | 1.41 | 1.29-1.53 | <0.001 |
| South | 4,288 (16.91) | 1,646 (20.55) | 1.34 | 1.22-1.47 | <0.001 |
| **Duration of diabetes (years)** |  |  |  |  |  |
| <10 | 13,459 (58.37) | 3,460 (47.94) | 1 |  |  |
| ≥10 | 9,599 (42.00) | 3,757 (52.06) | 1.24 | 1.16-1.33 | <0.001 |
| **BMI (kg/m^2^)** |  |  |  |  |  |
| Mean ± SD | 25.72 ± 4.75 | 25.86 ± 4.81 | 0.99 | 0.98-1.00 | 0.007 |
| **FPG (mg/dL)** |  |  |  |  |  |
| <100 | 2,123 (8.74) | 460 (6.04) | 1 |  |  |
| 100-199 | 20,429 (84.11) | 4,414 (57.93) | 1.11 | 0.99-1.25 | 0.08 |
| 200-299 | 1,568 (6.46) | 2,218 (29.11) | 5.22 | 4.56-5.98 | <0.001 |
| ≥300 | 167 (0.69) | 527 (6.92) | 9.65 | 7.7-12.09 | <0.001 |
| **Biguanide** |  |  |  |  |  |
| No | 6,012 (23.7) | 1,804 (22.52) | 1 |  |  |
| Yes | 19,351 (76.3) | 6,206 (77.48) | 1.47 | 1.36-1.60 | <0.001 |
| **Sulfonylurea** |  |  |  |  |  |
| No | 10,751 (42.39) | 2,813 (35.12) | 1 |  |  |
| Yes | 14,612 (57.61) | 5,197 (64.88) | 1.66 | 1.55-1.78 | <0.001 |
| **Thiazolidinediones** |  |  |  |  |  |
| No | 22,391 (88.28) | 6,718 (83.87) | 1 |  |  |
| Yes | 2,972 (11.72) | 1,292 (16.13) | 1.36 | 1.24-1.49 | <0.001 |
| **Insulin** |  |  |  |  |  |
| No | 21,580 (85.08) | 4,563 (56.97) | 1 |  |  |
| Yes | 3,783 (14.92) | 3,447 (43.03) | 4.31 | 3.99-4.66 | <0.001 |
| **Diabetic retinopathy** |  |  |  |  |  |
| No | 24,194 (95.39) | 7,440 (92.88) | 1 |  |  |
| Yes | 1,168 (4.61) | 570 (7.12) | 1.19 | 1.04-1.36 | 0.01 |

SD: standard deviation, UC: universal coverage, SSS: social security scheme, CSMBS: civil servant medical benefit scheme, BMI: body mass index, FPG: fasting plasma glucose, HbA1c: hemoglobin A1c, mg/dL: milligrams per deciliter, kg/m^2^: kilogram per square meter
