# Supplementary material for: Prognostic nomogram for uncontrolled type 2 diabetes using Thailand nation-wide cross-sectional studies
Source: PLoS One. 2024 Apr 10;19(4):e0298010. doi: 10.1371/journal.pone.0298010 (PMC11006157; doi:10.1371/journal.pone.0298010)
Supplement: S4 Table — AUC: Area under the curve, PPV: Positive predictive value, PPV: Negative predictive value, PLR: Positive likelihood ratio, NLR: Negative likelihood ratio. (DOCX) [file pone.0298010.s007.docx]

| **Table 4. Prediction performance of the nomogram for estimating uncontrolled diabetes.** | | |
| --- | --- | --- |
| **Prediction performance** | **Development** | **Validation** |
|  | **Group** | **Group** |
| **AUC (95%CI)** | 0.71 (0.70-0.72) | 0.70 (0.70-0.71) |
| **Cut-off value by Youden's index** | 0.23 | 0.23 |
| **Sensitivity, %** | 64.80 (63.60-65.90) | 69.70 (68.50-70.90) |
| **Specificity, %** | 77.00 (76.40-77.50) | 70.90 (70.20-71.60) |
| **PPV, %** | 46.70 (45.70-47.70) | 44.80 (43.70-45.80) |
| **NPV, %** | 87.50 (87.10-88.00) | 87.40 (43.70-45.80) |
| **PLR** | 2.81 (2.73-2.90) | 2.40 (2.33-2.47) |
| **NLR** | 0.46 (0.44-0.47) | 0.43 (0.41-0.44) |

AUC: Area under the curve, PPV: Positive predictive value, PPV: Negative predictive value, PLR: Positive likelihood ratio, NLR: Negative likelihood ratio
