# Supplementary material for: Prognostic nomogram for uncontrolled type 2 diabetes using Thailand nation-wide cross-sectional studies
Source: PLoS One. 2024 Apr 10;19(4):e0298010. doi: 10.1371/journal.pone.0298010 (PMC11006157; doi:10.1371/journal.pone.0298010)
Supplement: Attachment — Submitted filename: Comments_TL3.docx [file pone.0298010.s008.docx]

Re: MS No. PONE-D-22-19423

Prognostic Nomogram for Uncontrolled Type 2 Diabetes Using Thailand Nation-wide Cross-sectional Studies

PLOS ONE

Dear Reviewers,

Thank you for your concern regarding the above manuscript. We are grateful for the helpful comments made by the reviewer and have addressed them in detail below.

Reviewer #1

In Table 1. The FPG (mg/dL) was somehow wrong. It read mean+- SD equal to 36614.

**RESPONSE:** The recommended correction has been made. The data was rewritten into 153.53 ± 54.29 and 153.89 ± 55.76 in development and validation group, respectively.

In table 2-3. The percentage of each parameter in each HbA1c group should be in the sample group instead of in-between group because N in each group were a lot of difference. This would make it easier to see the difference. For example, in the duration of diseases, the authors should compare the percentage of individuals in the sample HbA1C group.

**RESPONSE:** The recommended correction has been made.

The references have to be adjusted. There are still errors in capitalization in the titles of references.

**RESPONSE:** The recommended correction has been made.

Regarding the prediction of uncontrolled T2DM, it was well accepted that high FPG is a strong predictor of uncontrolled diabetes. The problems these days are the individuals with good of borderline FPG who in fact have uncontrolled HbA1C (>8% or >9%). This is called "unaware uncontrolled diabetes". This group is in fact the real risk factors for developing complications. Secondly, the better and practical measurement to determine glucose homeostasis and short-term glycemic control which is similar to oral glucose tolerance test and glucose-insulin homeostasis is the “random postprandial glucose”. This measure is easy and available to everyone and every hospital; however, is not widely used. I would think this should mention in the discussion for possible practical solution.

```
RESPONSE: Thank you very much for your important issues to be added into the discussion section. We have added these paragraphs in the discission section.
“In countries with limited resources, the prediction of uncontrolled diabetes can be challenging. Some healthcare professionals may adjust a patient's medication based solely on their fasting plasma glucose (FPG) levels. However, some patients with acceptable or equivocal FPG levels may still have uncontrolled HbA1C levels (>8% or >9%).  This is called "unaware uncontrolled diabetes". In fact, it was the real risk factors for developing complications. (20–22) In addition to FPG, random postprandial glucose (RPG) testing is regarded as an effective method for evaluating short-term glycemic control and identifying individuals with uncontrolled diabetes who have normal fasting plasma glucose levels. (23)  Although RPG testing may not be widely used, it could be an alternative method for physicians to provide better individualized diabetes care instead of relying solely on HbA1c levels. This method may be used in those with uncontrolled diabetes identified by this nomogram.”
```

The authors mentioned in the “measurements” Section that lipid profiles were measured but they did not define the LDL levels in association with the lipid lowering medications if these results are in the control range. Please also define the definition of renal insufficiency, dyslipidemia, diabetes kidney diseases and others. For instance, if patients have LDL<100mg/dL with lipid lowering medication. Do they still consider dyslipidemia?

**RESPONSE:** The lipid profiles were not used in the analysis, so it is possible that they were not accounted for in the model.

We used the recorded database obtained from the ICD-10 code-based definition to identify renal insufficiency, dyslipidemia, diabetic kidney diseases, and other related conditions.

Reviewer #2: Authors have explored the feasibility of predictive nomogram for prognostic factors in type 2 diabetes. While they have come up with a long list of associated factors which help to identify poorly controlled diabetes, it is not practical to replace HbA1c testing on their so-called risk estimation with predictive nomograms as patients tend to get worse from low risk to high risk. Therefore, main message in this article is flawed however has some academic interest of read.

**RESPONSE:** I would propose that we build this model not to replace HbA1c testing, but rather to assist in the guidance of patients at risk of uncontrolled diabetes in resource-limited countries, such as Thailand, and especially in community hospitals with limited budgets. This model would facilitate frequent testing, but it must first be Hba1c-tested. We anticipate that this model will ultimately increase the ministry's budget for monitoring uncontrolled diabetes patients with hba1c testing more frequently than annually.

In the abstract, it is useful to define what the authors considered as uncontrolled diabetes (line 38)

**RESPONSE:** The recommended correction has been made.

Background: Line 55: prevalence increased to 31% seem to be very high, do they mean by 31%?

**RESPONSE:** I rewrote the sentence into:

Currently, in the Western Pacific Region, which includes Thailand, the prevalence of diabetes is 31%, or 212 million persons.

Line 58: change the word diabetics to diabetes.

**RESPONSE:** The recommended correction has been made.

Line 61: mortality including high glucose and diabetes: does not make sense, does it mean high glucose without confirmed diabetes? Please verify.

**RESPONSE:** I rewrote this sentence to clarify the term.

Line 70: change the word a diagnosis to higher after the onset of diabetes (cardiovascular disease risk goes up whether diagnosed or not, it is the onset, diagnosis maybe delayed)	I rewrote this sentence into

**RESPONSE:** Diabetes was associated with a two- to fourfold increased incidence of atherosclerotic cardiovascular disease compared to those without diabetes.

Line 79: change reduced to optimal HbA1c.

**RESPONSE:** The recommended correction has been made.

Line 90: Sentence does not read well, verify.

**RESPONSE:** I rewrote this sentence into

In order to promote improved glucose control, earlier insulin therapy initiation should be recommended if the HbA1c level is greater than 9.0%.

Line 92: Unable to grasp what is the relevance of MAP here, please verify this statement.

**RESPONSE:** I rewrote this sentence into

In addition, enhanced glycemic control through earlier initiation of insulin therapy could result in cardiovascular benefits, such as a reduction in blood pressure in patients with hypertension.

Line 103 type 2 diabetes patients not diabetics

**RESPONSE:** The recommended correction has been made.

Table 1: FPG, is this value of sample size or actual FPG? (36614, 30396)

**RESPONSE:** The recommended correction has been made. The data was rewritten into 153.53 ± 54.29 and 153.89 ± 55.76 in development and validation group, respectively.

Line 280 and everywhere change the word diabetic to diabetes.

**RESPONSE:** The recommended correction has been made.

Fig one is barely visible, I could not see. Unable to comment.

**RESPONSE:** I will add the high resolution of picture as your advice.

351: Paragraph does not read well: are the authors proposing testing only those who have met the nomogram prediction with HbA1c and use the cost saved to treat dyslipidemia? 75% of your population is already treated with statin as per data, you should not introduce new concepts unrelated to manuscript. Secondly, though your study and nomogram is based on cross sectional population, and has some predictive value, patients deteriorate and someone for instance has HbA1c of 7%, considered low risk according to the prediction model, there is no guarantee he/she will stay at 7%, that’s why periodic HbA1c testing is advocated.

**RESPONSE:** Thank you for your precious advice.

I rewrote this paragraph for clarify some misconception term.

Line 374: should be replaced by sugar and refined carbohydrates, excessive fat consumption.

**RESPONSE:** The recommended correction has been made.
