# Supplementary material for: Prognostic nomogram for uncontrolled type 2 diabetes using Thailand nation-wide cross-sectional studies
Source: PLoS One. 2024 Apr 10;19(4):e0298010. doi: 10.1371/journal.pone.0298010 (PMC11006157; doi:10.1371/journal.pone.0298010)
Supplement: Attachment — Submitted filename: Response to Reviewers_TL3.docx [file pone.0298010.s009.docx]

**Responses to Reviewers’ Comments**

Dear Reviewers,

Thank you very much for your valuable suggestions and comments on our manuscript. Your comments are immensely helpful in improving and revising our work.
We have carefully reviewed the comments and made corrections in line with your suggestions. The revised portions are highlighted in yellow in the paper. The main corrections in the paper and the responses to the reviewer's comments and remarks are as follows.

**1. Line 94-96 Thailand’s tight control…. Does not make sense. Is this Thailand’s guideline?**

Response: Thank you for your comments and concerns. This recommendation is in accordance with the Clinical Practice Guidelines for Diabetes 2017 in Thailand, which suggest that the follow-up care for diabetic patients depends on the severity of the disease and the chosen treatment approach. In the initial phase, patients should be scheduled for appointments every 1-4 weeks to provide them with knowledge about diabetes for self-management. Monitoring blood glucose levels and adjusting medication dosage to control blood sugar levels should be done according to the established targets within 3-6 months. Notably, HbA1c levels should be evaluated every 3-6 months or at least once a year. We trust this clarification addresses your concern.

**2. Abstract. Why use data 2018 for model building group and 2015 for validation group rather than the other way around?**

Response: Thank you for your valuable comment. Following your suggestion, we swapped the datasets for model building and validation. Surprisingly, this adjustment did not yield a significant difference in predicting the outcome of uncontrolled diabetes.

Our initial choice to use the 2018 dataset for model development was strategic. We aimed to capture the most recent trends in diabetes management and outcomes. Given the evolving nature of healthcare practices and patient demographics, utilizing a more recent dataset ensured that the nomogram incorporated the latest information.

The decision to use the 2015 dataset for validation was deliberate. This older dataset allowed us to assess the nomogram's performance on data not exposed during development. This approach evaluates the nomogram's generalizability across different time periods, ensuring its effectiveness despite potential shifts in healthcare practices or patient characteristics. We hope that these revisions and the improved text will be satisfactory.

**3. Line 167. More details are needed for the variables. For example, smoking habits can be smoker vs non-smoker but it can be also smoker, non-smoker, vs. prior smoker. Similar concern applies to other variables.**

Response: Thank you for highlighting the need for more details on the variables. The variable "smoking habits" in our dataset includes distinctions among current smokers, former smokers, and non-smokers. We appreciate your insight, and we will provide additional clarity in the methodology section to ensure transparency regarding the categorization of smoking habits.

**4. Line 172. For reproducibility, please also provide ICD-10 code for each disease.**

Response: Thank you for your valuable comments and suggestions. In line with your recommendations, we have incorporated the ICD-10 code into the revised manuscript within the measurements section. We trust that these revisions, coupled with the improved text, will meet your expectations.

**5. Line 180. Are all categorical variables binary?**

Response: Thank you for bringing this to our attention, and I appreciate your understanding. I have reviewed the sentence and consulted with our statistician to ensure clarity. The sentence in question has been revised to accurately convey that we conducted a comprehensive comparison of demographic characteristics between the development and validation groups. This involved assessing both categorical and continuous variables using the Chi-square test and independent two-sample t-test, respectively. The descriptive statistics are presented in Table 1.

I apologize for any confusion caused by the initial oversight, and I hope this clarification addresses your concern.

**6. Line 185. “the variance inflation factor variables were removed”. This doesn’t make sense. Also, what criteria was use? Please make sure to have statisticians involved in your study.**

Response: Thank you for your valuable comments and suggestions. We appreciate your careful review of our manuscript. In response to your query on Line 185 regarding the statement "the variance inflation factor variables were removed," we acknowledge the lack of clarity in our previous wording. We have revised this statement to provide a more lucid explanation. Specifically, we utilized the variance inflation factor (VIF) to address multicollinearity among the independent variables. Variables with elevated VIF values, indicating collinearity issues, were systematically identified and appropriately addressed in the model-building process.

Moreover, we would like to assure you that statisticians were actively involved in our study, contributing their expertise to the design, analysis, and interpretation of the statistical methods employed. We have emphasized this point in the revised manuscript to underscore the rigor and validity of our statistical approach.

We trust that these revisions, coupled with the improved text, will meet your expectations.

**7. Line 187. Please provide the criteria for backward selection.**

Response: Thank you for your valuable feedback. We appreciate the opportunity to address your concerns. Regarding the criteria for backward selection in our logistic regression analysis, we employed a significance level of *p*-value < 0.2 as the criterion for variable retention and removal. Specifically, at each step of the backward elimination process, the variable with the highest *p*-value exceeding 0.2 was iteratively removed until only variables with *p*-values below the significance threshold remained in the final model. This approach ensures that the selected variables in the multivariable logistic regression model are statistically significant contributors to the prediction of uncontrolled diabetes. We hope this clarification, coupled with the improved text, will meet your expectations.
